# Performance of D-dimer, cardiac troponin T, C-reactive protein, and NT-proBNP in prediction of long-term mortality in patients with suspected pulmonary embolism

**DOI:** 10.1093/ehjopen/oeae079

**Published:** 2024-09-20

**Authors:** Juha Kauppi, K E Juhani Airaksinen, Joonas Lehto, Jussi-Pekka Pouru, Juuso Saha, Petra Purola, Samuli Jaakkola, Jarmo Lehtonen, Tuija Vasankari, Markus Juonala, Tuomas Kiviniemi

**Affiliations:** Emergency Clinic, Turku University Hospital, Savitehtaankatu 1, 20540 Turku, Finland; Heart Centre, Turku University Hospital, PL 52, FI-20521 Turku, Finland; Clinical Medicine, University of Turku, PL 52, FI-20521 Turku, Finland; Heart Centre, Turku University Hospital, PL 52, FI-20521 Turku, Finland; Clinical Medicine, University of Turku, PL 52, FI-20521 Turku, Finland; Clinical Medicine, University of Turku, PL 52, FI-20521 Turku, Finland; Division of Medicine, Turku University Hospital, PL 52, FI-20521 Turku, Finland; Clinical Medicine, University of Turku, PL 52, FI-20521 Turku, Finland; Division of Medicine, Turku University Hospital, PL 52, FI-20521 Turku, Finland; Heart Centre, Turku University Hospital, PL 52, FI-20521 Turku, Finland; Clinical Medicine, University of Turku, PL 52, FI-20521 Turku, Finland; Emergency Clinic, Turku University Hospital, Savitehtaankatu 1, 20540 Turku, Finland; Heart Centre, Turku University Hospital, PL 52, FI-20521 Turku, Finland; Clinical Medicine, University of Turku, PL 52, FI-20521 Turku, Finland; Division of Medicine, Turku University Hospital, PL 52, FI-20521 Turku, Finland; Heart Centre, Turku University Hospital, PL 52, FI-20521 Turku, Finland; Clinical Medicine, University of Turku, PL 52, FI-20521 Turku, Finland

**Keywords:** Pulmonary embolism, Mortality, Prognosis, NT-proBNP, C-reactive protein, FIDD, cTNT

## Abstract

**Aims:**

Pulmonary embolism (PE) is a common and potentially life-threatening condition requiring emergent diagnostic work-up. Despite wide use of biomarkers, little is known how they predict long-term prognosis of patients evaluated for suspected PE.

**Methods and results:**

We sought to assess the predictive performance of N-terminal pro-brain natriuretic peptide (NT-proBNP), C-reactive protein, fibrin D-dimer (FIDD), and cardiac troponin T (cTnT) in patients who underwent computed tomography pulmonary angiography (CTPA) for clinical suspicion of PE. The analysis involved 1001 patients, with 222 (22.2%) receiving a PE diagnosis at index imaging. Mean ages of patients with and without PE were 65.0 ± 17.1 and 64.5 ± 17.7 years, respectively. Median follow-up time was 3.9 years (interquartile range 2.9–4.9). Mortality was relatively high among both patients with and without documented PE (24.8% vs. 31.7%, *P* = 0.047). In patients with PE, only elevated NT-proBNP > 1000 ng/L and C-reactive protein > 50 mg/L levels at hospital admission were associated with higher mortality in an adjusted Cox regression model, but receiver operating characteristic (ROC) analysis showed no improved prediction compared to clinical variables. Among patients without PE, elevated NT-proBNP > 1000 ng/L, C-reactive protein > 10 mg/L, cTnT > 50 ng/L, and FIDD > 1.0 mg/L all predicted mortality. In an ROC analysis among patients without PE, models including NT-proBNP, cTnT, or C-reactive protein provided improved predictive performance.

**Conclusion:**

Patients evaluated for clinical suspicion of PE have high long-term mortality. Commonly used biomarkers provide long-term prognostic value in patients without PE. Given the relatively young age, it is vital to identify these high-risk patients and perform differential diagnosis work-up for alternative life-threatening conditions, and manage them as appropriate.

## Graphical Abstract

**Graphical Abstract:**
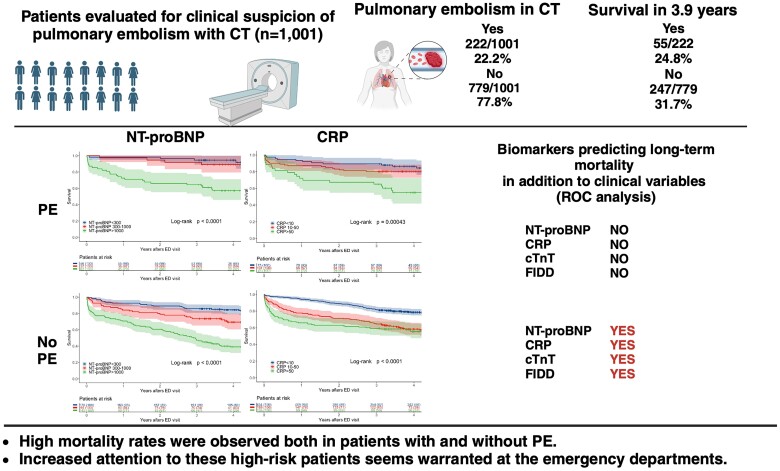

## Introduction

Pulmonary embolism (PE) is a common and potentially life-threatening differential diagnosis option at the emergency department. Various biomarkers, such as N-terminal pro-brain natriuretic peptide (NT-proBNP), C-reactive protein, fibrin D-dimer (FIDD), and cardiac troponin T (cTnT) are routinely used to guide clinical decision-making and assess in-hospital and short-term risk and prognosis. Usefulness of biomarkers in the long-term prognostic assessment in patients with suspected PE is not known at the emergency room setting.

NT-proBNP and troponins have been shown to predict short-term mortality in patients with PE.^1,2^ Elevated troponin levels associate with adverse outcome in various cardiac and non-cardiac populations,^3–6^ but there is lack of data how these markers predict long-term mortality. C-reactive protein was found to be useful for predicting outcomes, such as early death and bleeding in patients with venous thromboembolism.^7,8^ Similarly, high CRP is associated with recurrence risk of venous thromboembolism in cancer patients after discontinuation of anticoagulation with no effect on mortality.^8^ Fibrin D-dimer predicts cancer-associated thrombosis,^9^ but there is insufficient evidence regarding its relevance in other patient populations.

We sought to assess the predictive performance of NT-proBNP, C-reactive protein, FIDD, and cTnT in a cohort of patients who underwent computed tomography pulmonary angiography (CTPA) for clinical suspicion of PE.

## Methods

The study included 1001 patients undergoing CTPA in the emergency unit during the study period as described in our previous study.^10^ Data were retrospectively collected from the medical records over the period of 1 January 2014 to 31 December 2016. The patient was considered eligible in the study if CTPA was performed during the emergency unit visit with no specific exclusion criteria. If a patient had several CTPAs over the study period, only the first CTPA was included. The Ethics Committee of the Wellbeing Services County of Southwest Finland waived consenting the study subjects, due to the retrospective observational nature of the study.

Trained research personnel collected data through a structured electronic case report form, encompassing patients’ medical history, ongoing medications, clinical examination findings, laboratory results, and reports from CTPA imaging. The data collectors adhered to predefined criteria, consulting with senior team members in cases of uncertainty to arrive at final decisions when coding the data. The CTPA followed the Turku University Hospital clinical CTPA protocol, with interpretation performed by radiologists from the emergency clinic. Additionally, we secured approval to utilize the death certificates from Statistics Finland, the official national statistics agency of Finland. The information obtained from the death certificates included the date of death and the causes of death for patients who were deceased between 1 January 2014 and 31 December 2019. In our previous article from this study group, we demonstrated the manifested symptoms in the study population. Dyspnoea was the most frequent symptom with 190 (85.6%) and 576 (73.9%) patients with and without PE experiencing shortness of breath.^10^

The cTnT and NT-proBNP values were analysed with electrochemiluminescence immunoassay (ECLIA, sandwich principle, Elecsys cTnT hs and Elecsys NT-proBNP II, Li-heparin plasma, Roche Diagnostics). The analytical range was 3–10 000 (ng/L) and 5–35 000 (ng/L) for cTnT and NT-proBNP. The C-reactive protein values were analysed with particle-enhanced immunoturbidimetric assay (Tina-quant CRP IV, Roche Diagnostics). The analytical range was <1–700 (mg/L). The FIDD values were analysed with particle-enhanced immunoturbidimetric assay {D-DI2 [Tina-quant FIDD Gen. 2 (2015-10, V5)], citrated plasma, Roche Diagnostics}. The analytical range was 0.2–21.6 mg/L.

### Cut-off values for biomarkers

The minimum cut-off value for FIDD was set at 1.0 mg/L, with no need for applying age-adjusted values. That was based on data safely excluding PE in patients with low-intermediate pre-test likelihood of PE.^11^ The upper limit for the cut-off value was determined by considering studies that have investigated short-term prognosis, with many setting the maximum cut-off within the range of 1.5–5 and significant proportion falling between 3 and 4.^12^

The minimum threshold for CRP was set at 10 mg/L. The upper limit was set at 50 (mg/L) based on study where C-reactive protein levels of 48–50 (mg/L) were identified as an optimal cut-off value for predicting short-term mortality in PE patients.^7,13^

Age-independent NT-proBNP cut-off level of <300 (ng/L) was selected as it rules out effectively acute decompensated heart failure (HF) (99% negative predictive value).^14^ The upper limit cut-off level of 1000 (ng/L) was chosen based on PARADIGM-HF substudy.^15^

Numerous research have studied cTnT cut-off levels in short-term prognosis of PE patients, varying from 10 to 100 ng/L.^1,16^ In this study, the established lower and upper cut-off thresholds for cTnT were 50 and 100 (ng/L) used.

### Statistical analysis

SPSS (version 29.0.0.0, 64-bit edition) and R softwares (version 2023.09.1+494) were used in statistical analysis. The continuous variables were reported as mean ± standard deviation. Categorical variables were reported as counts and percentages. Kaplan–Meier graphs with log rank test were used for conducting survival analysis. The Mantel–Cox test was used to calculate the *P*-values for survival analysis. Receiver operating characteristic (ROC) analysis and area under the curve (AUC) values were used to illustrate the diagnostic ability of NT-proBNP, FIDD, C-reactive protein, and cTnT. *P* < 0.05 were considered as significant.

## Results

A total of 1001 patients underwent CTPA, and 22.2% (222) of them had PE at index imaging. Baseline characteristics of the study population are presented in *Table 1*. Mean ages of patients with and without PE were 65.0 ± 17.1 and 64.5 ± 17.7 years, respectively. At median of 3.9 years (interquartile range 2.9–4.9) follow-up, mortality rate was 24.8% (55/222) vs. 31.7% (247/779) in patients with and without PE diagnosis (*P* 0.047), respectively. Detailed co-morbidities of the study population have been described in the previous study.^10^

**Table 1:** Baseline characteristics of the study population (*N* = 1001)

Male	412 (41.2)
Age	64.8 ± 17.5
Prior deep vein thrombosis/pulmonary embolism	137 (13.7)
Prior decompensated heart failure	98 (9.8)
Prior obstructive coronary disease	150 (15.0)
Prior myocardial infarction	80 (8.0)
Prior atrial fibrillation	116 (11.6)
NT-proBNP (ng/L)	2528.6 (5911.7)
cTnT (ng/L)	43.7 (136.6)
CRP (mg/L)	30.5 (55.1)
FIDD (mg/L)	3.1 (4.4)

Data are presented as count (%) or mean ± SD.

NT-proBNP, N-terminal pro-brain natriuretic peptide; cTnT, cardiac troponin T; CRP, C-reactive protein; FIDD, fibrin D-dimer.

The underlying causes of death in patients with and without PE diagnosis are presented in *Table 2*. The most common causes for death in patients with PE were cancer and dementia, whereas 3.2% (7/222) died of PE. Among those without PE diagnosis at index imaging, most common reasons for death were cancer and coronary artery disease, while 0.5% (4/779) died of PE during the follow-up. We also present the main causes of death in patients with and without PE within 30-day follow-up (see Supplementary material online, *Tables S2* and *S3*).

**Table 2:** The underlying and contributing causes of death (%) in patients with and without pulmonary embolism in computed tomography pulmonary angiography during 3-year follow-up

	PE N = 55	No PE N = 247
Cancer	15 (27.2)	78 (31.6)
Obstructive coronary disease	5 (9.1)	48 (19.4)
Pulmonary embolism	7 (12.7)	4 (1.6)
Infection	2 (3.6)	14 (5.7)
Dementia	12 (21.8)	13 (5.3)
Chronic pulmonary disease	3 (5.5)	22 (8.9)
Accident/suicide	5 (9.1)	9 (3.6)
Hypertension	1 (1.8)	12 (4.9)
Diabetes	1 (1.8)	4 (1.6)
Stroke	1 (1.8)	8 (3.2)
Renal failure	0	1 (0.4)
Other reason	3 (5.5)	25 (10.1)

**Figure 1:**
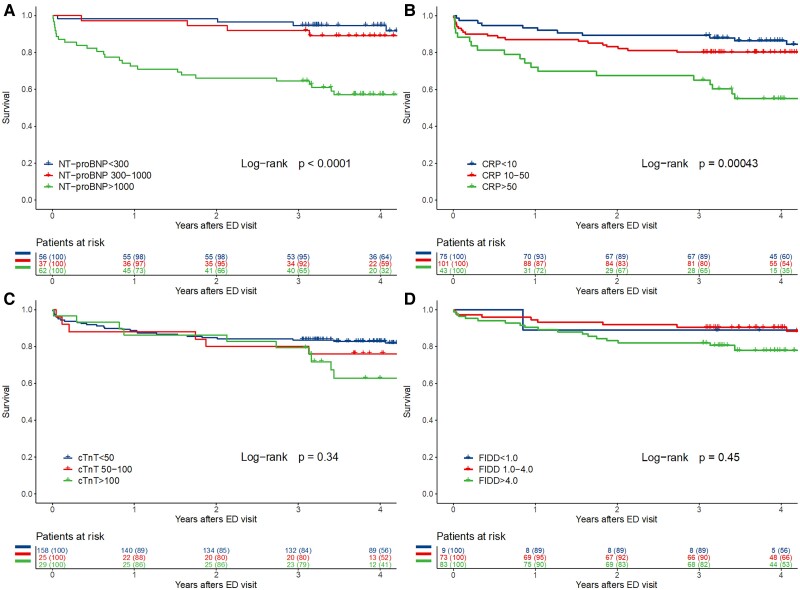
Kaplan–Meier survival analysis of patients with pulmonary embolism in computed tomography pulmonary angiography according to (*A*) N-terminal pro-brain natriuretic peptide classes, (*B*) C-reactive protein, (*C*) cardiac troponin T, and (*D*) fibrin D-dimer.

**Figure 2:**
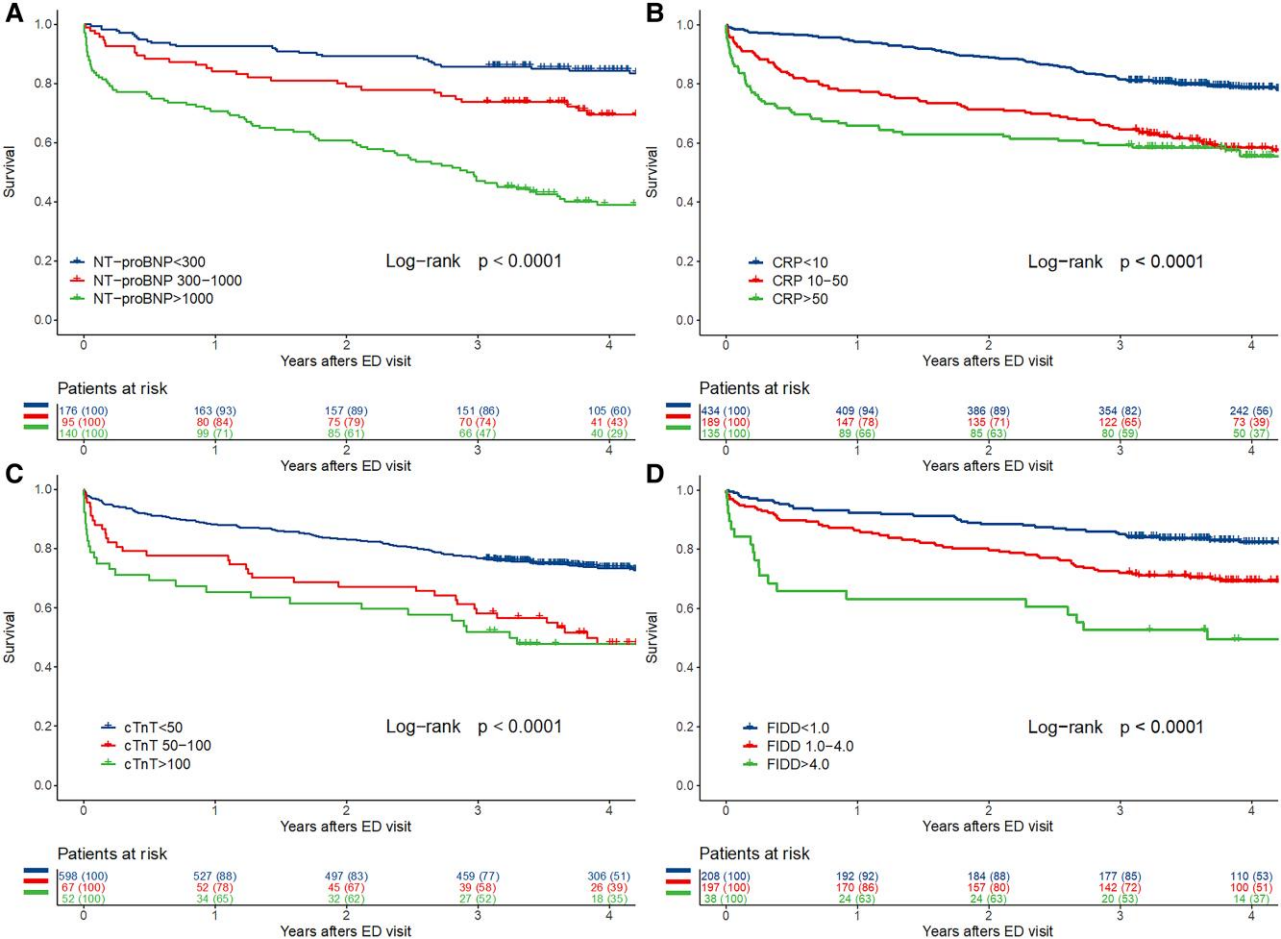
Kaplan–Meier survival analysis of patients without pulmonary embolism in computed tomography pulmonary angiography according to (*A*) N-terminal pro-brain natriuretic peptide classes, (*B*) C-reactive protein, *C*) cardiac troponin T, and (*D*) fibrin D-dimer.

Biomarkers were measured from patients on clinical grounds; NT-proBNP in 155/222 (69.8%) and 411/779 (52.8%), cTnT 212 (95.5%) and 717 (92.0%), C-reactive protein 219 (98.6%) and 758 (97.3%), and FIDD 165 (74.3%) and 443 (56.9%) of patients with and without PE. Different biomarker levels in predicting later mortality are presented in *Figures 1* and *2* stratified according to PE status. Cox multivariable regression analyses adjusted with age, sex, and cancer diagnosis are presented in *Table 3* and using continuous biomarker levels in Supplementary material online, *Table S4*. When biomarkers were tested separately, elevated C-reactive protein > 50 mg/L and NT-proBNP > 1000 ng/L were associated with worse long-term survival in patients with PE. In patients without PE, those with NT-proBNP > 1000 ng/L, cTnT > 50 ng/L, C-reactive protein > 10 mg/L, or FIDD > 4.0 mg/L had worse survival compared to those with lower levels of each biomarker.

**Table 3:** Cox multivariable regression analysis adjusted with age, sex, and cancer diagnosis and various biomarker

Variable	PE			No PE		
	Exp	95% CI	*P* value	Exp	95% CI	*P* value
Age	1.1	1.0–1.1	<0.001	1.0	1.0–1.1	<0.001
Sex (male)	1.5	0.74–3.0	0.26	1.4	1.0–1.9	0.05
Cancer diagnosis	3.0	1.5–6.3	0.003	2.3	1.6–3.2	<0.001
NT-proBNP < 300 ng/L			Ref			Ref
NT-proBNP 300–1000 ng/L	0.67	0.19–2.3	0.52	1.7	1.0–2.8	0.05
NT-proBNP > 1000 ng/L	3.1	1.2–6.3	0.02	3.5	2.2–5.4	<0.001
Age	1.1	1.1–1.1	<0.001	1.1	1.0–1.1	<0.001
Sex (male)	1.2	0.67–2.1	0.54	1.3	1.0–1.7	0.04
Cancer diagnosis	2.3	1.2–4.3	0.008	1.8	1.4–2.4	<0.001
cTnT < 50 ng/L			Ref			Ref
cTnT 50–100 ng/L	0.90	0.36–2.2	0.82	1.9	1.3–2.7	<0.001
cTnT > 100 ng/L	1.1	0.50–2.3	0.87	2.4	1.6–3.6	<0.001
Age	1.1	1.1–1.1	<0.001	1.1	1.0–1.1	<0.001
Sex (male)	1.4	0.81–2.5	0.22	1.4	1.1–1.8	0.02
Cancer diagnosis	2.2	1.3–4.0	0.006	1.9	1.5–2.6	<0.001
CRP < 10 mg/L			Ref			Ref
CRP 10–50 mg/L	1.5	0.74–2.9	0.27	2.2	1.6–2.9	<0.001
CRP > 50 mg/L	2.6	1.3–5.2	0.007	3.0	2.1–4.1	<0.001
Age	1.1	1.1–1.2	<0.001	1.0	1.0–1.1	<0.001
Sex (male)	1.5	0.72–3.2	0.28	1.5	1.0–2.1	0.04
Cancer diagnosis	1.9	0.85–4.1	0.12	1.8	1.2–2.8	0.005
FIDD < 1.0 mg/L			Ref			Ref
FIDD 1.0–4.0 mg/L	0.55	0.07–4.5	0.58	1.5	0.97–2.2	0.07
FIDD > 4.0 mg/L	0.49	0.06–3.9	0.50	2.9	1.7–5.0	<0.001

**Figure 3:**
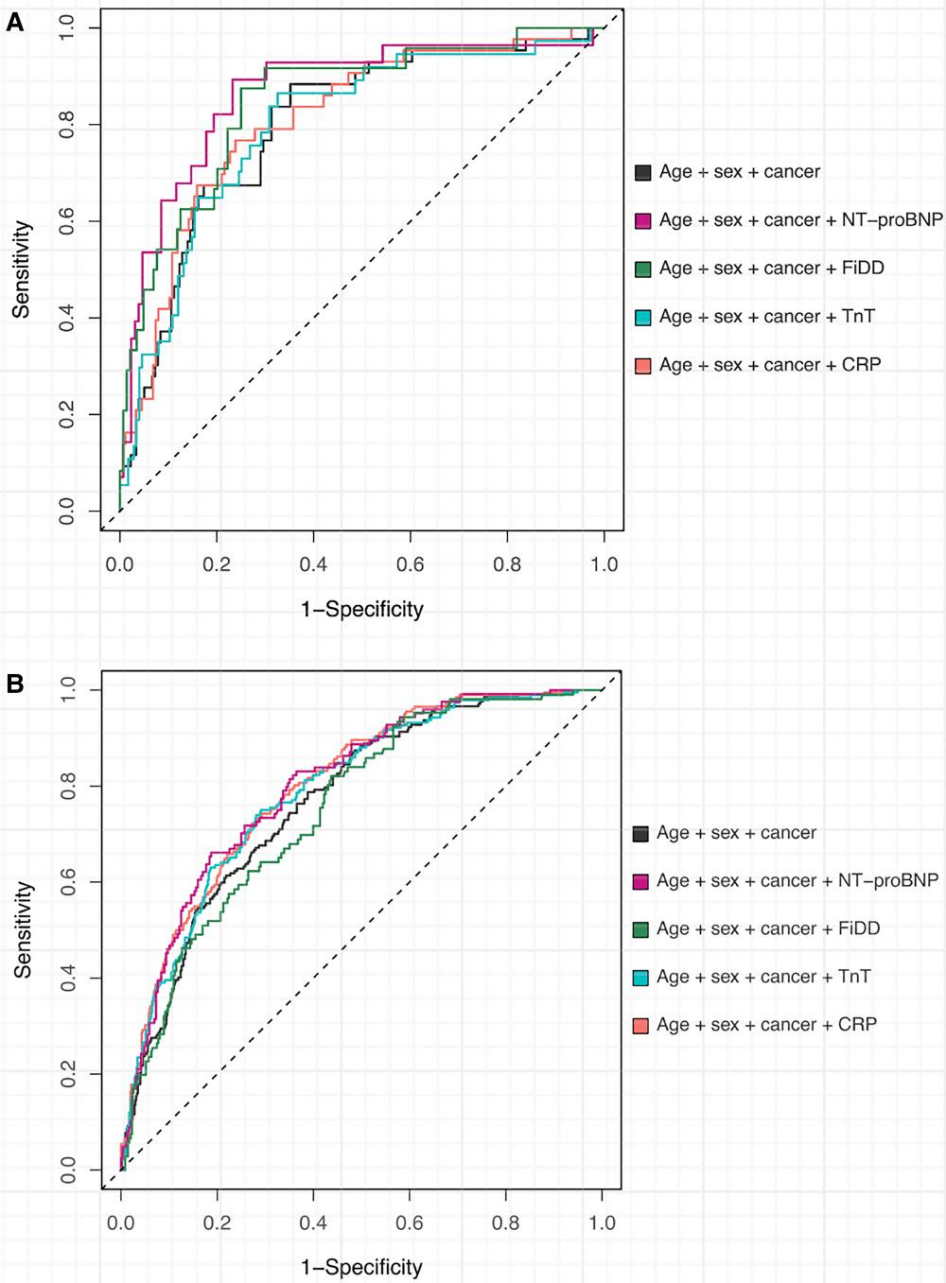
Receiver operating characteristics analysis of patients with (*A*) and without (*B*) pulmonary embolism in computed tomography pulmonary angiography and levels of N-terminal pro-brain natriuretic peptide, C-reactive protein, fibrin D-dimer, and cardiac troponin T.

The ROC curves of each biomarker in patients with or without PE to predict 3-year mortality are shown in *Figure 3*. Among patients with PE, only the NT-proBNP tended to improve predictive power when compared to a baseline model including age, sex, and cancer, with AUC values of 86.7 (95% CI 78.7–94.8) and 81.8 (95% CI 72.9–90.6, *P* = 0.071, respectively, *N* = 157).

In patients without PE, addition of NT-proBNP [AUC 80.2 (95% CI 75.8–84.6) vs. 76.1 (95% CI 71.3–80.8), *N* = 412], CRP [AUC 80.0 (95% CI 76.6–83.3) vs. 77.1 (95% CI 73.6–80.7), *N* = 759), and cTnT [AUC 78.9 (95% CI 75.3–82.5) vs. 77.0 (95% CI 73.3–80.7), *N* = 718] provided improved predictive performance compared to a model including age, sex, and cancer (*P* < 0.05 for all comparisons).

Area under the curve analysis for 3-year mortality in patients with and without PE using continuous cTnT is presented in the Supplementary material online, *Figure S1*. The AUC values were 0.66 and 0.73 in these groups, respectively.

## Discussion

Biomarkers are routinely used in the risk stratification of patients suspected for PE. The main findings of this study were that biomarker levels at the emergency clinic have distinctive prognostic value in patients with or without PE. In patients with PE, only CRP levels >50 mg/L and NT-proBNP levels > 1000 ng/L predicted poor long-term survival. In contrast, in patients without PE, elevated levels of all biomarkers (cTnT, CR-reactive protein NT-proBNP, and FIDD) predicted worse prognosis. As expected, patients with FIDD < 1 mg/L, C-reactive protein < 10 mg/L, NT-proBNP < 300 ng/L, or cTnT < 50 ng/L had the best prognosis both in patients with and without PE.

Patients with suspected PE at the emergency department have relatively poor survival. The mortality rates were 24.8% and 31.7% in patients with and without PE at the median follow-up of 3.9 years in spite of the relatively young age of the patients (57.2% and 54.3% patients with and without PE older than 65 years).^10^ It is noteworthy that the prognosis was worse for patients without PE compared to those with PE. This can be explained by some critically ill patients with shortness of breath requiring evaluation to exclude PE. Additionally, if the underlying cause was not identified, appropriate treatment was not initiated. This contrasts with patients who were diagnosed with PE, as it is most often a treatable condition.

Patients with or without PE and high NT-proBNP (>1000 ng/L) had poor outcome in this study, which is consistent with previous studies.^2^ Pathophysiology for elevated NT-proBNP levels in patients with PE include increase in pulmonary vascular resistance and afterload of the right ventricle (RV) followed by RV dilatation, leading to the release of NT-proBNP. Individuals with chronically elevated levels of NT-proBNP, especially with RV dysfunction,^17^ are likely to have diminished cardiac reserve, when confronted with another concurrent illness such as PE. For those without PE and elevated NT-proBNP levels, acute decompensated, HF, and atrial fibrillation are the most common differential diagnostic options.

The most common differential diagnosis options for those without PE and elevated cTnT include acute coronary syndrome, atrial fibrillation, and acute decompensated HF. In accordance with NT-proBNP, cTnT also possesses the capability to recognize low-risk PE patients and exhibits a strong negative predictive value (97–100%) for in-hospital deaths.^18^ Although diverse range of cut-off values were used, a meta-analysis concluded that the elevated troponin levels in PE patients are linked to a 5-fold risk of short-term mortality from all causes.^1^ In the present study, the long-term prognostic value was observed only in patients without PE.

In our Supplementary material, we provide AUC analysis for 3-year mortality in patients with and without PE using continuous cTnT levels. The AUC analysis suggests a moderate discriminatory ability of continuous cTnT in predicting mortality between these patient groups. However, patients with cTnT levels < 50 ng/L had a better prognosis, regardless of PE status. Due to the ambiguity in the literature regarding prognostic cut-offs for cTnT, these current cut-offs were selected.^1,16^

A prior study concluded that CRP could safely exclude PE independently or when combined with clinical probability assessment.^19^ Although C-reactive protein is not included in the diagnostic strategy of the ESC guidelines for the diagnosis and management of acute PE,^20^ C-reactive protein may have prognostic value in PE.^7,13^ The present study supports the long-term prognostic value of C-reactive protein both in patients with and without PE. Therefore, if C-reactive protein is elevated without obvious underlying reasons, additional diagnostic measures seem justified.

Numerous other conditions including infections and cancer may elevate FIDD besides PE.^21^ Cancer is also a major risk factor for VTE as patients with malignancies are more likely to develop VTE even in the absence of other thrombogenic factors. These patients are prone to both thrombotic and haemorrhagic events.^9^ Furthermore, cancer-associated thrombosis is a major cause of morbidity and mortality in cancer patients.^22^ Therefore, clinicians frequently encounter difficulties in clinically ruling out PE without CTPA in cancer patients with elevated FIDD levels, especially when the clinical suspicion for PE is otherwise low. Previously Himeno *et al*. suggested that FIDD has prognostic value in cancer-associated thrombosis. However, there is lack of evidence concerning its significance in other patient groups. In the present study, FIDD had prognostic value only in patients without PE but not in patients with PE. One plausible explanation is that PE is frequently triggered by a temporary factor. After addressing this factor and initiating PE treatment, the long-term prognosis of the patient is often good in spite of the initial high FIDD value. On the other hand, administering appropriate treatment for VTE may not necessarily lead to a reduction in FIDD levels among cancer patients.

Patient with PE had significantly better outcome compared to those without PE. This difference may be attributed to the treatable nature of PE. The main causes of death clearly indicate that patients with malignant neoplasm and those with obstructive coronary disease are well represented in this study group. This distribution also reflects the emergency clinic patient population.

This study needs to interpret with some limitations. This was a retrospective, single-centre study, and laboratory results and the follow-up data were collected from the medical records by the trained personnel. We collected data on initiated anticoagulant therapies, thrombolytic treatments, and mechanical interventions, such as thrombectomies, but we did not include other therapies. Despite the conclusiveness of laboratory results, age, sex, and CTPA results, obtaining a comprehensive medical history can be challenging using patient records. This challenge stems from instances where patients have a history of multiple medical conditions and numerous healthcare encounters and a potential for undetected confounders exists. Finally, the size of the cohort with PE was quite small with limited predictive power.

## Conclusions

The mortality is high in patients with suspected PE both in those with and without confirmation of PE. Routinely measured biomarkers measured on admission have prognostic value especially among patients without PE. Increased attention to these high-risk patients seems warranted at the emergency departments.

## Lead author biography


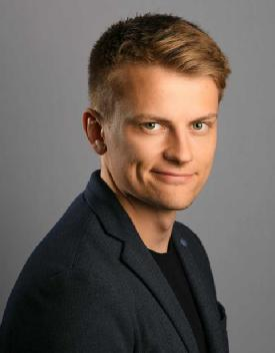


Dr Juha Kauppi is a specialized emergency physician. He earned his medical degree from Turku University Medical School and completed his specialization in emergency medicine at Turku University Hospital. With over seven years of experience, Dr Kauppi has worked extensively in emergency departments and across various medical disciplines, including anesthesiology, critical care, prehospital emergency care, pediatrics, and internal medicine. Throughout his career, Dr. Kauppi has consistently aimed to advance emergency medical care. His expertise in managing a wide range of acute medical conditions and procedures, from heart attacks to severe injuries, has made him a trusted and respected figure in the medical community. Currently, Dr. Kauppi is completing his PhD while residing in Milan, Italy. Outside of his professional life, he enjoys hiking, spending time with his family, and is a passionate padel player.

## Supplementary Material

oeae079_Supplementary_Data

## Data Availability

Data are available upon a reasonable request excluding variables falling under GDPR regulation.
