# Supplementary material for: Performance of D-dimer, cardiac troponin T, C-reactive protein, and NT-proBNP in prediction of long-term mortality in patients with suspected pulmonary embolism
Source: Eur Heart J Open. 2024 Sep 20;4(5):oeae079. doi: 10.1093/ehjopen/oeae079 (PMC11467688; doi:10.1093/ehjopen/oeae079)
Supplement: oeae079_Supplementary_Data [file oeae079_supplementary_data.docx]

Supplement figure 1. AUC analysis of continuous cTnT levels over a 3-year follow-up period, categorized by mortality in patients with (A) and without (B) PE.


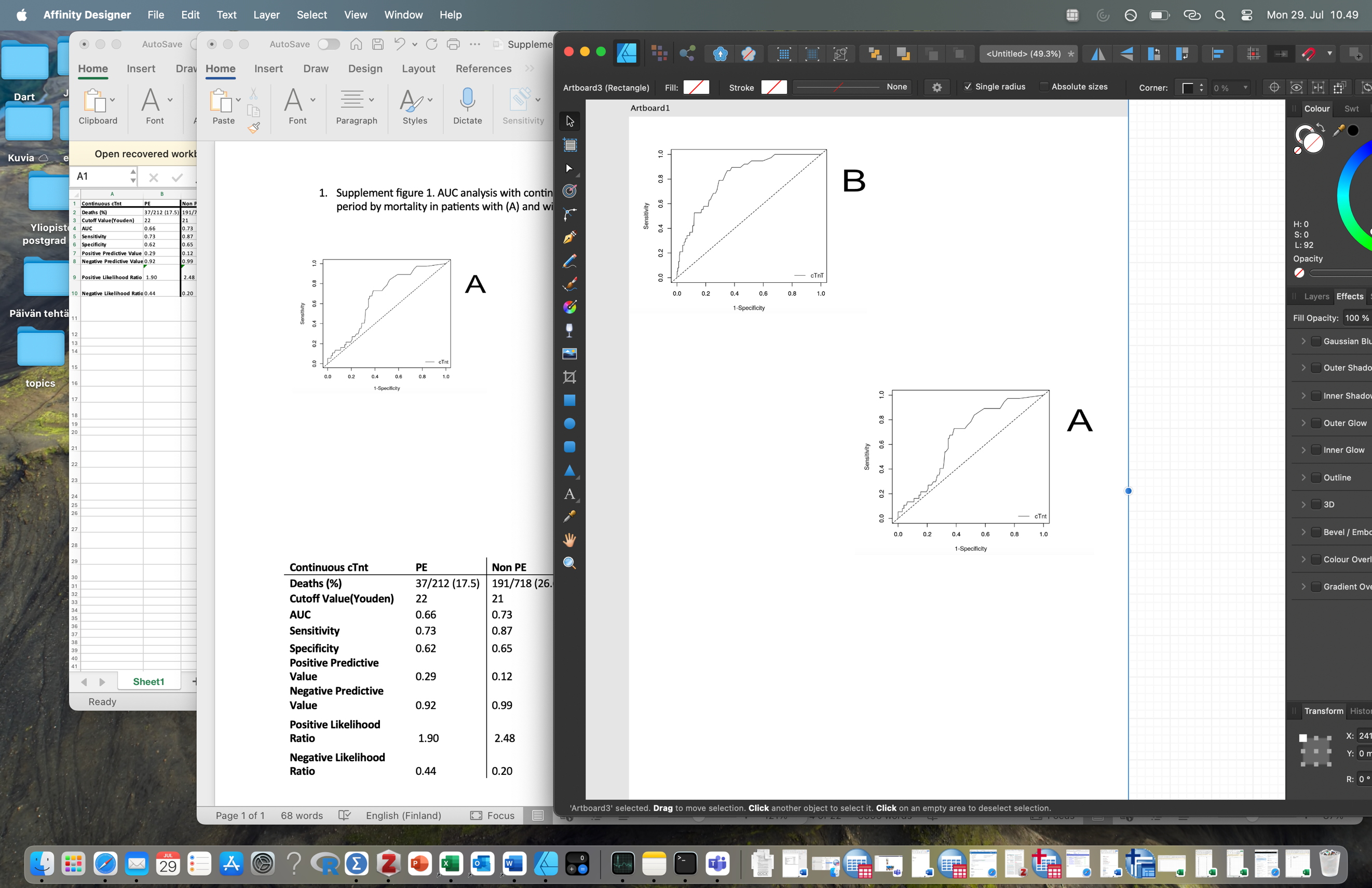

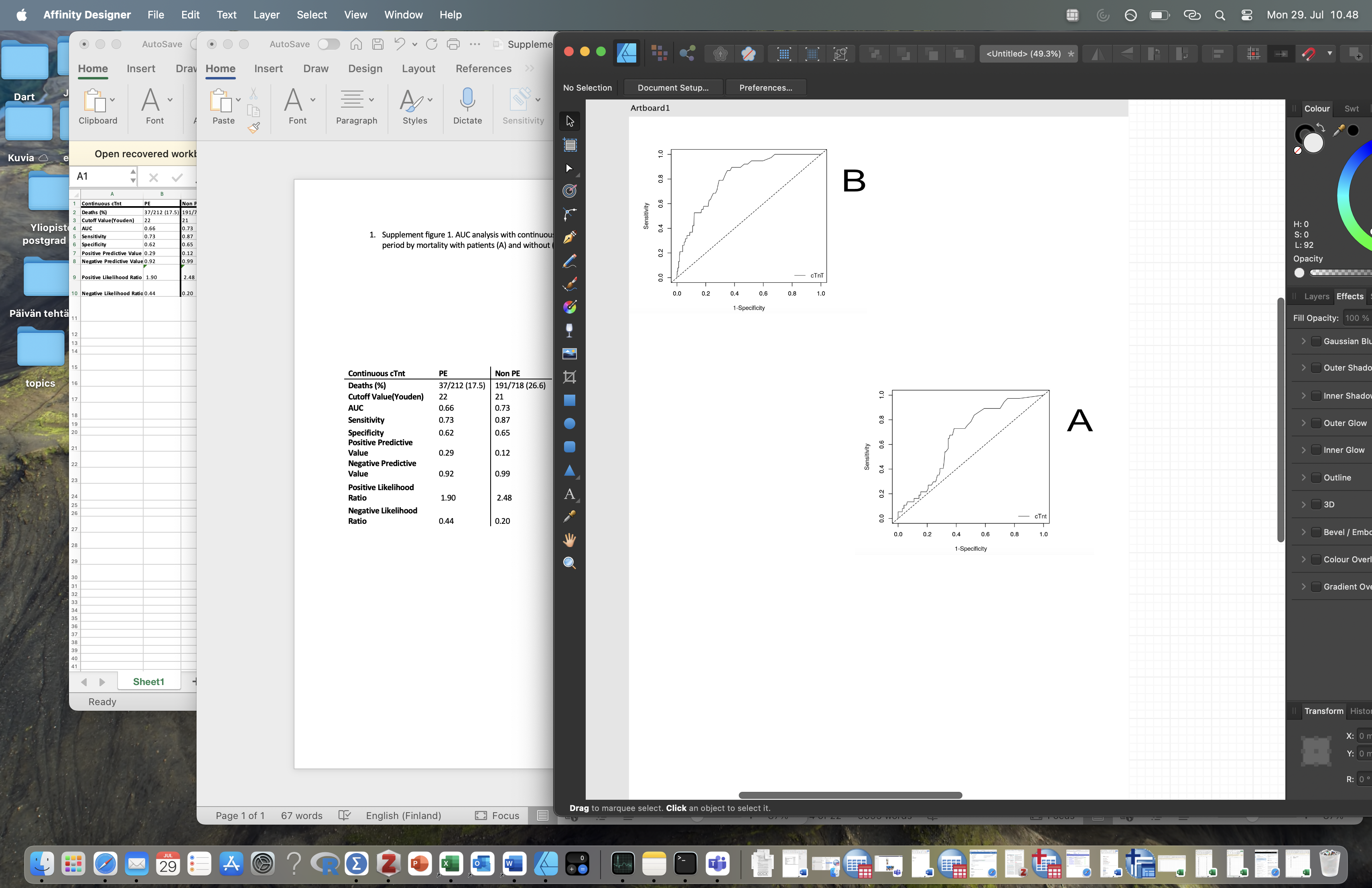


| **Continuous cTnt** | **PE** | **Non PE** |
| --- | --- | --- |
| **Deaths (%)** | 37/212 (17.5) | 191/718 (26.6) |
| **AUC** | 0.66 | 0.73 |
| **Sensitivity** | 0.73 | 0.87 |
| **Specificity** | 0.62 | 0.65 |
| **Positive Predictive Value** | 0.29 | 0.12 |
| **Negative Predictive Value** | 0.92 | 0.99 |
| **Positive Likelihood Ratio** | 1.90 | 2.48 |
| **Negative Likelihood Ratio** | 0.44 | 0.20 |
|  |  |  |

Supplementary Table 1. Most frequent diagnoses for patients, who underwent CTPA without having PE in the emergency clinic.

| **Diagnoses (ICD10)** | **N (%)** |  |
| --- | --- | --- |
| **R06 Dyspnea** | 111 (14.2%) |  |
| **J18 Pneumonia** | 64 (8.2) |  |
| **I50 Heart failure** | 63 (8.1) |  |
| **R07 Pain in throat and chest** | 31 (4.0) |  |
| **R55 Syncope and collapse** | 26 (3.3) |  |
| **J44-J45 Asthma and COPD** | 25 (3.2) |  |
| **I21 Acute myocardial infarction** | 20 (2.6) |  |
| **I48 Atrial fibrillation and flutter** | 17 ( 2.2) |  |
| **Z03 Medical observation and evaluation of suspected diseases and conditions ruled out** | 13 (1.7) |  |
|  |  |  |
| **I46 Cardiac arrest** | 10 (1.3) |  |
| **Total (n=779)** | 380 (48.7) |  |

Supplementary Table 2. Main causes of death in patients without PE identified in index imaging within 30-day follow-up. Data was gathered from the death certificates.

| **Main causes of death** | **N (%)** |
| --- | --- |
| **C16- C92 Malignant neoplasm** | 10 (25.0) |
| **I25 Chronic ischemic heart disease** | 9 (22.5) |
| **J84 Interstitial pulmonary disease** | 3 (7.5) |
| **I11 Hypertensive heart disease** | 2 (5.0) |
| **I21 Acute myocardial infarction** | 2 (5.0) |
| **Total (n=40)** | 26 (57.8) |

Supplementary Table 3. Main causes of death in patients with PE identified in index imaging within 30-day follow-up. Data was gathered from the death certificates.

| **Main causes of death** | **N (%)** |
| --- | --- |
| **C34-C83 Malignant neoplasm** | 5 (35.7) |
| **I26 Pulmonary Embolism** | 2 (14.3) |
| **G30 Alzheimer´s disease** | 2 ( 14.3) |
| **I25 Chronic ischemic heart disease** | 1 (7.1) |
| **E11 Type 2 diabetes mellitus** | 1 (7.1) |
| **I42 Cardiomyopathy** | 1 (7.1) |
| **I63 Cerebral infarction** | 1 (7.1) |
| **W01 Fall on same level slipping, tripping and stumbling** | 1 (7.1) |
| **Total (n=14)** | 14 (100) |

Supplementary Table 4. Cox multivariable regression analysis adjusted with age, sex and cancer diagnosis and various biomarkers as continuous variables.

|  |  | **PE** |  |  | **No PE** |  |
| --- | --- | --- | --- | --- | --- | --- |
| **Variable** | **Exp** | **95%-CI** | **P value** | **Exp** | **95%-CI** | **P value** |
| **Age** | 1.1 | 1.0-1.1 | <0.001 | 1.0 | 1.0-1.1 | <0.001 |
| **Sex (male)** | 1.4 | 0.72-2.7 | 0.33 | 1.5 | 1.2-2.2 | 0.005 |
| **Cancer diagnosis** | 2.8 | 1.3-5.9 | 0.007 | 2.0 | 1.4-2.9 | <0.001 |
| **NT-proBNP (ng/L)** | **1.0** | **1.0-1.0** | **0.006** | **1.0** | **1.0-1.0** | **<0.001** |
| **Age** | 1.1 | 1.0-1.1 | <0.001 | 1.1 | 1.0-1.1 | <0.001 |
| **Sex (male)** | 1.2 | 0.65-2.1 | 0.59 | 1.4 | 1.1-1.8 | 0.02 |
| **Cancer diagnosis** | 2.3 | 1.2-4.3 | 0.008 | 2.0 | 1.5-2.6 | <0.001 |
| **cTnT (ng/L)** | 1.0 | 1.0-1.0 | 0.25 | **1.0** | **1.0-1.0** | **<0.001** |
| **Age** | 1.1 | 1.1-1.1 | <0.001 | 1.1 | 1.0-1.1 | <0.001 |
| **Sex (male)** | 1.5 | 0.85-2.6 | 0.17 | 1.4 | 1.1-1.9 | 0.006 |
| **Cancer diagnosis** | 2.2 | 1.3-4.0 | 0.006 | 2.1 | 1.6-2.8 | <0.001 |
| **CRP (mg/L)** | **1.0** | **1.0-1.0** | **0.005** | **1.0** | **1.0-1.0** | **0.005** |
| **Age** | 1.1 | 1.1-1.2 | <0.001 | 1.0 | 1.0-1.1 | <0.001 |
| **Sex (male)** | 1.5 | 0.72-3.1 | 0.27 | 1.5 | 1.1-2.2 | 0.02 |
| **Cancer diagnosis** | 1.8 | 0.80-4.0 | 0.14 | 1.8 | 1.2-2.9 | 0.005 |
| **FIDD (mg/L)** | 1.0 | 0.94-1.0 | 0.65 | **1.1** | **1.0-1.1** | **<0.001** |
